# Supplementary material for: Wide-field magnetometry using nitrogen-vacancy color centers with randomly oriented micro-diamonds
Source: Sci Rep. 2022 Oct 26;12:17997. doi: 10.1038/s41598-022-22610-5 (PMC9606006; doi:10.1038/s41598-022-22610-5)
Supplement: Supplementary file 1 — Supplementary Information. [file 41598_2022_22610_MOESM1_ESM.pdf]

Wide-field magnetometry using nitrogen-vacancy color centers with randomly oriented micro-diamonds

Saravanan Sengottuvel[1,*], Mariusz Mrózek[1], Mirosław Sawczak[2], Maciej J. Głowacki[3], Mateusz Ficek[3], Wojciech Gawlik[1], and Adam M. Wojciechowski[1,*]

[1]Institute of Physics, Jagiellonian University in Krakow, 11 Łojasiewicza St., 30-348 Kraków, Poland
[2]Szewalski Institute of Fluid-Flow Machinery, Polish Academy of Sciences, 14 Fiszera St., 80-231 Gdańsk, Poland
[3]Gdańsk University of Technology, 11/12 G. Narutowicza St., 80-233 Gdańsk, Poland

Supplementary information

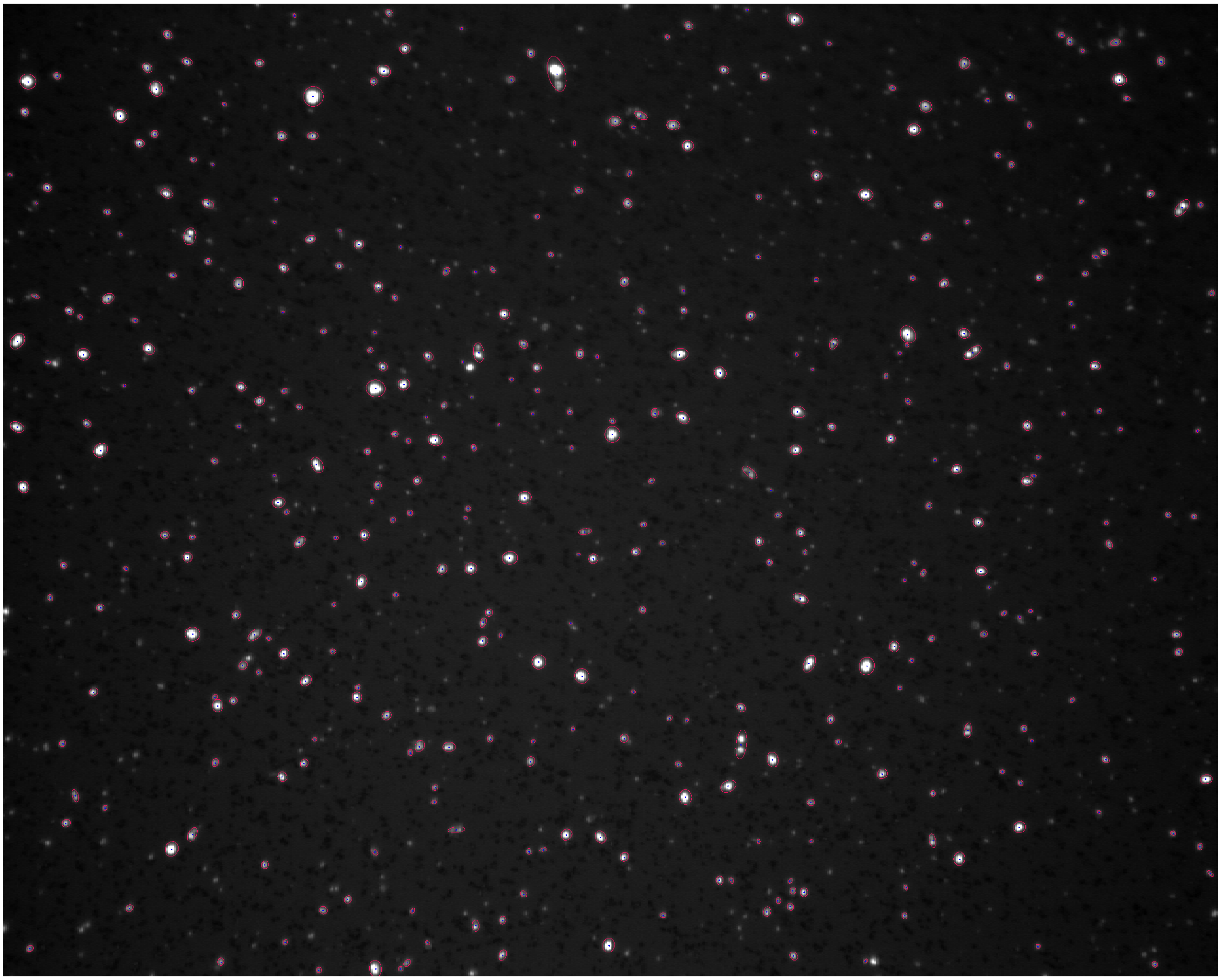

Figure S1. Bright fluorescent diamond spots identified by the automated ODMR reconstruction algorithm from a single image obtained by the camera. The pixel region is encircled in red with XY coordinates of the spots marked in blue.

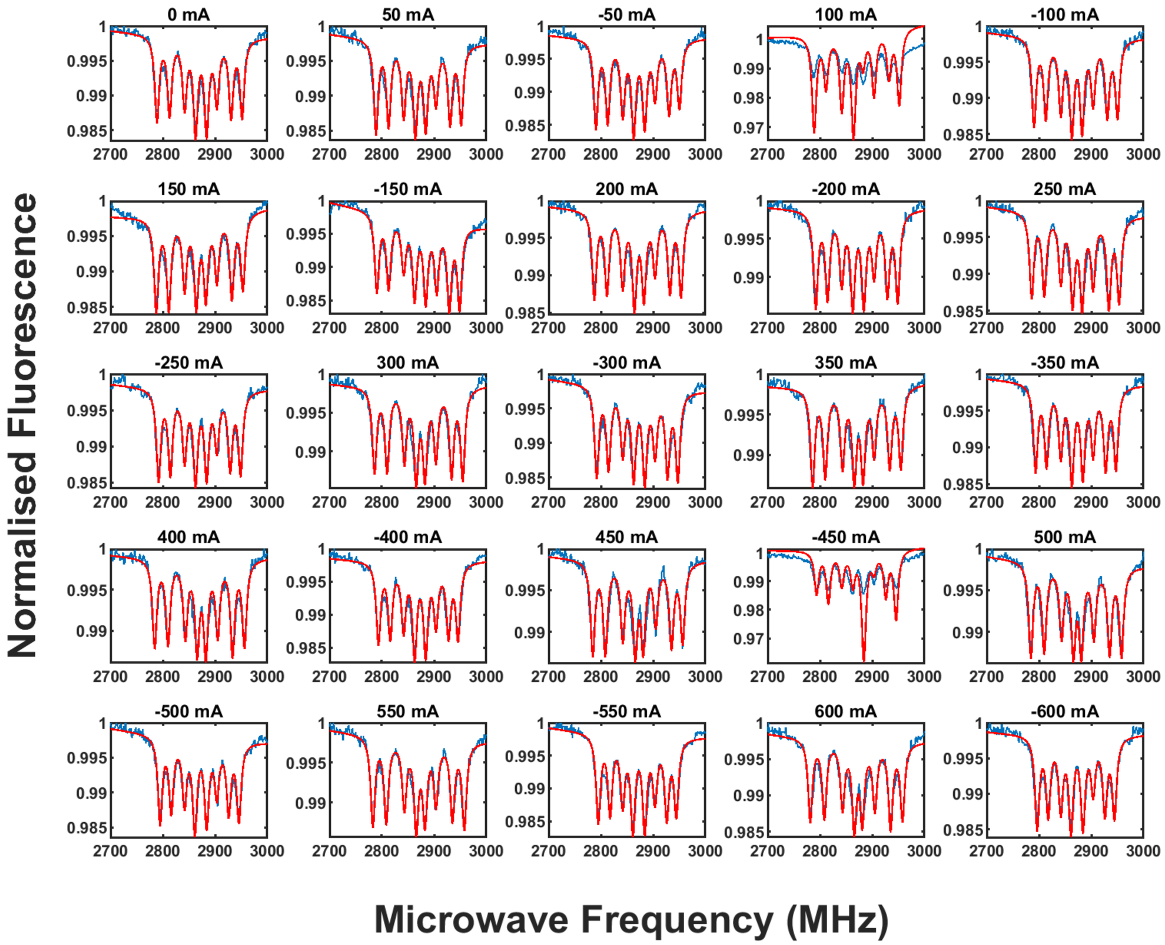

Figure S2. An example of multi-Lorentzian curve fitting to the ODMR spectra retrieved from a single diamond spot using the automated algorithm for different current values.

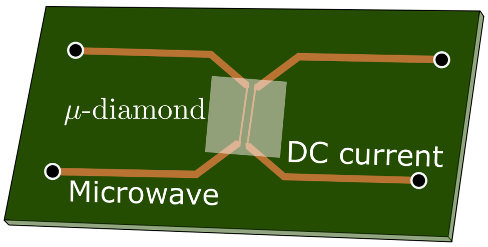

Figure S3. Schematic of the microwave and current striplines with a thin layer of micro-diamond powder deposited on a glass coverslip.
